# Dopamine Transporter Genetic Reduction Induces Morpho-Functional Changes in the Enteric Nervous System

**DOI:** 10.3390/biomedicines9050465

**Published:** 2021-04-24

**Authors:** Silvia Cerantola, Valentina Caputi, Gabriella Contarini, Maddalena Mereu, Antonella Bertazzo, Annalisa Bosi, Davide Banfi, Dante Mantini, Cristina Giaroni, Maria Cecilia Giron

**Affiliations:** 1 Department of Pharmaceutical and Pharmacological Sciences, University of Padova, 35131 Padova, Italy; silvia.cerantola@unipd.it (S.C.); valekap@gmail.com (V.C.); maddamereu@gmail.com (M.M.); antonella.bertazzo@unipd.it (A.B.); 2 Department of Poultry Science, University of Arkansas, Fayetteville, AR 72704, USA; 3 Department of Biomedical and Biotechnological Sciences, University of Catania, 95131 Catania, Italy; gabriella.contarini@unict.it; 4 Department of Medicine and Surgery, University of Insubria, 21100 Varese, Italy; a.bosi@uninsubria.it (A.B.); d.banfi3@uninsubria.it (D.B.); cristina.giaroni@uninsubria.it (C.G.); 5 IRCCS San Camillo Hospital, 30126 Venice, Italy; dante.mantini@ospedalesancamillo.net or; 6 Motor Control and Neuroplasticity Research Group, KU Leuven, 3000 Leuven, Belgium

**Keywords:** dopamine transporter, enteric nervous system, small intestine, neuromuscular contractility, confocal microscopy

## Abstract

Antidopaminergic gastrointestinal prokinetics are indeed commonly used to treat gastrointestinal motility disorders, although the precise role of dopaminergic transmission in the gut is still unclear. Since dopamine transporter (DAT) is involved in several brain disorders by modulating extracellular dopamine in the central nervous system, this study evaluated the impact of DAT genetic reduction on the morpho-functional integrity of mouse small intestine enteric nervous system (ENS). In DAT heterozygous (DAT^+/−^) and wild-type (DAT^+/+^) mice (14 ± 2 weeks) alterations in small intestinal contractility were evaluated by isometrical assessment of neuromuscular responses to receptor and non-receptor-mediated stimuli. Changes in ENS integrity were studied by real-time PCR and confocal immunofluorescence microscopy in longitudinal muscle-myenteric plexus whole-mount preparations (). DAT genetic reduction resulted in a significant increase in dopamine-mediated effects, primarily via D1 receptor activation, as well as in reduced cholinergic response, sustained by tachykininergic and glutamatergic neurotransmission via NMDA receptors. These functional anomalies were associated to architectural changes in the neurochemical coding and S100β immunoreactivity in small intestine myenteric plexus. Our study provides evidence that genetic-driven DAT defective activity determines anomalies in ENS architecture and neurochemical coding together with ileal dysmotility, highlighting the involvement of dopaminergic system in gut disorders, often associated to neurological conditions.

## 1. Introduction

Gene deficiency or reduction of dopamine transporter (DAT) gene is a valuable model for investigating DAT dysfunction, which is usually involved in transmitter imbalances in psychiatric and neurodegenerative diseases, (e.g., addictive disorders, attention-deficit/hyperactivity disorder, schizophrenia, Parkinson’s disease), frequently associated to gastrointestinal disorders [1,2]. DAT operates as the site of action for a variety of addictive drugs and therapeutic reuptake inhibitors which determine increased dopamine levels in both central and peripheral nervous systems, leading to a higher dopaminergic activity. Indeed, DAT knockout (DAT^−/−^) mice do not efficiently clear extracellular dopamine and display a generally overactive dopaminergic state, representing a well-known model to examine alterations in dopaminergic homeostasis in response to persistent hyperdopaminergic tone. Potentiation of dopaminergic neurotransmission was demonstrated also in the large intestine of DAT^−/−^ mice, further supporting the hypothesis that dopamine exerts a physiologically important restraint on gut motility [3]. In the gastrointestinal tract, dopamine induces several effects, depending on specific receptor subtypes, on the region and gut layer involved [4,5]. Five dopamine receptors have been genetically identified and clustered into two main families: the D1-like family, comprising the D1 and D5 receptors, and the D2-like family, comprising D2, D3, and D4 receptors. Apart from the D4 receptor, that is exclusively expressed on the mucosal layer, all the other dopaminergic receptors are involved in the modulation of intestinal contractility [4]. Dopaminergic neurons have been found in rodent and human enteric nervous system (ENS) and are characterized by the presence of the tyrosine hydroxylase (TH) enzyme and DAT as well as by the absence of dopamine β-hydroxylase enzyme [4,6]. D1-like receptors are localized on the soma and nerve endings impinging on the intestinal wall and mucosa, whereas D2 receptor has been found solely in neurons [7]. A transgenic heterozygous mouse for DAT gene (DAT^+/−^) has been developed to evaluate the pathophysiological importance of the transporter. Although the majority of studies have been carried out on DAT^−/−^, DAT^+/−^ animals represent an interesting preclinical model with peculiar phenotypic, developmental and behavioral characteristics, due to the two-fold reduction of DAT, which leads to a proportional increase of extracellular dopamine levels [8,9,10]. In this perspective, DAT^+/−^ mice may represent a useful model to investigate in more detail the importance of the dopaminergic transmission in the modulation of the intestinal neuromuscular function. Indeed, the possibility to elucidate the role of DAT in the gut as well as its influence on neurotransmitter pathways within the ENS, may strengthen the causal relationship between development of brain disorders associated to gastrointestinal comorbidities, such as addictive disorders, attention-deficit/hyperactivity disorder, schizophrenia, Parkinson’s disease and dopaminergic system dysregulation. This is all the more interesting since psychostimulants, used as addictive drugs, or several antidopaminergic drugs, prescribed as prokinetics [11] or antipsychotics [12], show relevant gastrointestinal symptoms [13].

Based on this evidence, we sought to characterize the role of DAT genetic reduction in controlling small bowel neuromuscular function. To this end we have investigated the consequences of DAT hypofunction on the morphofunctional integrity of ENS and the related signaling pathways. Our data may lend novel and useful hints translatable into innovative pharmacological strategies for patients with psychiatric and neurological disorders, clinically associated to bowel disorders.

## 2. Materials and Methods

### 2.1. Animals

All animal care and experimental procedures were approved by the Animal Care and Use Ethics Committee of the University of Padova and by the Italian Ministry of Health (authorization number: 41451.N.NRD, 11 January 2020) and were performed in compliance with national and EU guidelines for the handling and use of experimental animals. Animal studies are reported in agreement with the ARRIVE guidelines [14]. 

Female DAT heterozygous (DAT^+/−^; 14 ± 3 weeks old) mice and sex- and age-matched wild type (WT; DAT^+/+^) C57BL6/J mice were housed in ventilated cages (IVC; four animals per cage) at the conventional animal facility of the Department of Pharmaceutical and Pharmacological Sciences, University of Padova, Italy, under controlled environmental conditions (temperature 21 ± 1 °C; relative humidity 60–70%) with a regular 12/12 h light/dark cycle, free access to standard laboratory chow and tap water. Original DAT^−/−^ mice were backcrossed with C57BL6J mice for at least 10 generations [15]. The breeding scheme was based on mating DAT^+/−^ heterozygous males with DAT^+/+^ female mice to ensure no changes in maternal behavior. PCR analysis of tail DNA was performed for mouse genotyping, as previously described [9,16]. All the following experimental procedures were blind to genotype. A total of 50 mice (i.e., 25 mice for each transgenic group) were used to carry out the following experiments.

### 2.2. In Vitro Contractility Studies

Contractility experiments were performed as previously described [17,18]. Briefly, 1-cm-distal ileum segments, proximal to the ileocecal junction, were isolated and mounted along the longitudinal axis in organ baths containing 10 mL of oxygenated (95% O_2_ and 5% CO_2_) and heated (37 °C) Krebs solution (in mM: NaCl 118, NaHCO_3_ 25, C_6_H_12_O_6_ 11, KCl 4.7, CaCl_2_∙_2_H_2_O 2.5, MgSO_4_∙1.2, K_2_HPO_4_ 1.2). Changes in mechanical activity of ileum segments were recorded by isometric transducers (World Precision Instruments, Berlin, Germany) connected to a quad bridge amplifier and PowerLab 4/30 data acquisition system using LabChart6 software (ADInstruments, Besozzo, VA, Italy) [19].

After 45 min equilibration, small intestine segments were stretched passively to a 0.5 g initial tension and then brought to their optimal point of length-tension relationship using 1 μM carbachol (CCh). Ileal preparations were precontracted with 10 μM CCh for 2 min until stable responses were obtained and then subjected to 1 μM isoprenaline (non-selective β adrenoreceptor agonist). Ileal segments were treated with CCh to study cholinergic-mediated responses or subjected to electrical field stimulation (EFS) to evaluate neuronally-mediated contractions or exposed to 60 mM KCl to elicit depolarization-induced contraction. Concentration–effect curves to CCh (0.001–100 μM) were obtained cumulatively and plotted into a nonlinear regression model (fitted to a sigmoidal equation) to calculate EC50 and maximal tension (Emax) values. EFS was performed at increasing frequencies (0–50 Hz; 1 ms pulse duration; 10 s pulse-trains, 40 V) using platinum electrodes connected to an S88 stimulator (Grass Instrument, Quincy, MA, USA) [20]. In order to generate non-noradrenergic, non-cholinergic transmitter (NANC) nerve stimulation, both guanethidine (1 μM) and atropine (1 μM) were added to Krebs solution and ileal tissues were allowed to equilibrate for 1 h prior to EFS stimulation. Under NANC conditions, the effects of the non-selective NOS inhibitor L-NAME (100 μM; preincubation time  =  20 min) was recorded on 10 Hz-EFS-induced on-relaxations. In order to evaluate the influence of tachykininergic neurotransmission on ileal contraction, the 10 Hz EFS-mediated tachykininergic off-response was assessed in presence of 100 μM L-NAME and 10 μM L732138 (NK1 receptor antagonist) under NANC conditions. To assess the glutamatergic response, ileal preparations were suspended in a Mg^++^-free Krebs solution and treated with the glutamatergic agonist N-methyl-D-aspartate (NMDA) 100 μM and 1 mM [21]. To determine changes in dopaminergic response, concentration-response curves to dopamine were constructed in a non-cumulative approach (0.1–300 μM) on basal tone [4]. In order to evaluate the contribution of D1 and D2 receptor, a submaximal dose of dopamine (30 μM) was tested after in the presence of SCH-23390 (10 μM; D1 receptor antagonist) or sulpiride (10 μM, D2 receptor antagonist) in absence or presence of 10 μM CCh-precontracted isolated ileal segments. To assess the involvement of dopamine on neuromuscular response, 4 Hz EFS stimulation was performed in presence of 10 μM dopamine with or without SCH-23390 (10 μM) or sulpiride (10 μM) or L-NAME (100 µM) or MRS2500 (10 μM; P2Y1 receptor antagonist). Contractile responses were expressed as gram tension/gram dry tissue weight of ileal segments and ileal relaxation was calculated as AUC and normalized per g dry tissue weight and expressed as a percentage.

### 2.3. Immunohistochemistry on Ileal Whole Mount Preparations

Immunohistochemistry studies were performed as previously described [17]. To assess changes in ENS architecture, fresh isolated distal ileum 10 cm-segments were gently flushed with warm (37 °C) Krebs solution, to remove any luminal content, and then ileal segments were rinsed with phosphate buffer solution (PBS) and exposed to fixative solution (4% paraformaldehyde in PBS) for 2 h at room temperature. After three 15 min-washes in PBS, ileal segments were cut in 0.5 cm-pieces opened along the mesenteric border and placed as a flat sheet to the bottom of Sylgard-coated dishes with the mucosal side down. Using a dissecting microscope, tissues were separated into two layers: the outer musculature with adhering serosa and the submucosa/mucosa. The circular muscle was removed to yield whole mounts of longitudinal muscle with attached myenteric plexus (LMMPs). LMMPs preparations were gently stretched and pinned down on the bottom of Sylgard-coated dishes and washed in PBT (PBS with 0.3% Triton X-100) for 45 min with gentle shaking. For substance P (SP) immunostaining, the excised tissue was initially incubated for 4 h in Krebs solution, bubbled with 95% O_2_ and 5% CO_2_ containing colchicine (0.1 g·L^−1^) in order to enhance the immunofluorescence of SP^+^ neurons [20]. After blocking nonspecific sites with PBT containing 5% BSA for 1.5 h at room temperature, LMMPs were incubated overnight at room temperature with the primary antibodies (Table 1) diluted in PBT and 5% BSA. The following day, LMMPs preparations were washed and incubated for 2 h at room temperature with respective secondary antibodies (Table 1) diluted in PBT and 5% BSA. After three subsequent 15 min washes with PBT, LMMPs preparations were mounted on glass slides using mounting solution (Citifluor™ Mountant Solution AF1; Società Italiana Chimici, Rome, Italy) and stored at −20 °C in the dark until analysis. Negative controls were obtained by incubating sections with isotype-matched control antibodies at the same concentration as primary antibody and/or pre-incubating each antibody with the corresponding control peptide (final concentration as indicated by manufacturer’s instructions).

### 2.4. Confocal Image Acquisition and Analysis

Images were acquired using a Zeiss LSM 800 confocal imaging system (ZEN 2.3 (blue edition); Oberkoken, Germany) equipped with an oil-immersion 63× objectives (NA 1.4). Z-series images (25 planes for LMMP whole mount preparations) of 1024 pixels × 1024 pixels were captured and were processed as maximum intensity projections. All microscope settings were kept constant for all images. Fluorescence intensity (density index) of GFAP, S100β, D1 receptor, choline acetyltransferase (ChAT), substance P was assessed for each antigen by capturing 20 images per mouse, as previously reported [17]. The intensity of staining for each antibody was expressed as the density index of labelling normalized per myenteric ganglion area and was reported as mean ± SEM. In ileal myenteric plexus, total neuronal population analysis was performed on LMMP preparations from five animals per group by counting HuC/D^+^ cells in 10 randomly images per mouse. To evaluate the distribution of nitrergic neurons in ileal myenteric plexus, the number of nNOS^+^ enteric neurons was blindly counted in 10 randomly chosen images per mouse. Areas of myenteric ganglia, digitized by capturing 20 fields per preparation, were measured by tracing boundaries around stained cell somas (HuC/D). The total number of HuC/D^+^ or nNOS^+^ neurons was recorded in each image and normalized per myenteric ganglion area, as previously described [17,20].

### 2.5. RNA Isolation and Quantitative RT-PCR for Glun1 Subunit Of NMDA Receptor

Total RNA was extracted from small intestine wall segments after removing the mucosa with TRIzol (Invitrogen; Monza, Italy), as described by Bistoletti et al. (2020) [22]. cDNA was obtained by retrotranscribing 2.5 µg of total RNA using the High-Capacity cDNA synthesis kit (Applied Biosystems, Milan, Italy). Quantitative RT-PCR was performed on the Abi Prism 7000 real-time thermocycler (Applied Biosystems, Milan, Italy) with Power Sybr Green Universal PCR Master Mix (Applied Biosystems, Milan, Italy) according to the manufacturer’s instructions. Primers for GluN1 and β-actin, which was used as housekeeping gene, were designed using Primer Express software (Applied Biosystems, Milan, Italy). Primer sequences were 5′-CAGGAGCGGGTAAACAACAGCAAC-3′, 5′-GCAGCCCCACCAGCAGCCACAGT-3′, for GluN1 and ACCAGAGGCATACAGGGACA, CTAAGGCCAACCGTGAAAAG for β-actin. For each RT-PCR analysis primers were used at a final concentration of 500 nM. Primers were designed to have a similar amplicon size and similar amplification efficiency as required for applying the 2-∆∆Ct method to compare gene expression in DAT^+/−^ group with respect to values obtained in WT [23]. β-actin was used as housekeeping gene. Experiments were performed at least five times for each different preparation.

### 2.6. Materials

Unless otherwise specified, all chemicals were obtained from Sigma–Aldrich (Milan, Italy) and were of the highest commercially available analytical grade. PFA and Citifluor™ Mountant Solution AF1 were purchased from Electron Microscopy Sciences (Società Italiana Chimici, Rome, Italy), and Triton-X-100 was obtained from Applichem (Milan, Italy).

### 2.7. Data and Statistical Analysis

The data and statistical analysis in this study comply with the recommendations on experimental design and analysis in pharmacology [24]. All the experiments were analyzed by investigators blinded to the genotypes. All data are expressed as mean ± SEM. The distribution of data was tested with the Shapiro–Wilk normality test. Differences between the experimental groups were assessed using paired or unpaired Student’s *t*-test, one-way analysis of variance (ANOVA), followed by Newman–Keuls post hoc test for multiple comparison, or two-way ANOVA followed by Bonferroni’s multiple comparison test for post hoc analysis, or the non-parametric Mann–Whitney’s U-test for independent variables, using GraphPad Prism v.8.4 (San Diego, CA, United States). The differences between groups were considered significant at *p* < 0.05; *n* values indicate the number of animals. Post hoc tests were run only if F achieved *p* < 0.05 and there was no significant variance inhomogeneity.

## 3. Results

### 3.1. DAT Heterozygosis Influences Dopamine-Mediated Response of Small Intestine

**Figure 1:**
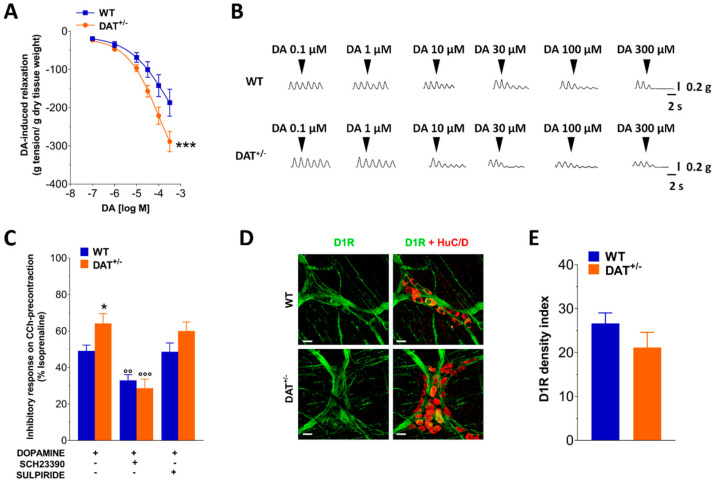
DAT hypofunction affects dopaminergic neuromuscular response. (**A**) Inhibitory concentration–response curves to dopamine (DA) on spontaneous contractile activity of isolated ileal preparations from WT and DAT^+/−^ mice. (**B**) Representative tracings of responses induced by increasing dopamine (DA) concentration of in WT and DAT^+/−^ preparations. (**C**) 30 µM dopamine-induced relaxation of CCh-precontracted ileal preparations with or without SCH23390 or sulpiride in isolated ileal segments of WT and DAT^+/−^ mice. (**D**) Representative confocal microphotographs showing the distribution of D1R (green, marker for D1 receptor) and HuC/D (red, pan-neuronal marker) and (**E**) D1R density index in LMMP preparations of WT and DAT^+/−^ mice. Scale bars = 22 μm. Data are reported as mean ± SEM. * *p* < 0.05, *** *p* < 0.001 vs. WT mice; °° *p* < 0.01, °°° *p* < 0.001 vs. respective control in absence of antagonist. *n* = 5 mice/group.

Isolated ileal preparations from WT and DAT^+/−^ mice exhibited comparable spontaneous contractile activity in terms of frequency (0.52 ± 0.0034 cps vs. 0.52 ± 0.0060 cps, respectively, *n* = 20 per genotype) and amplitude (0.23 ± 0.021 g vs. 0.28 ± 0.021 g, respectively, *n* = 20 per genotype). Since dopamine is an endogenous modulator of intestinal motility in the mouse gastrointestinal tract through the engagement of gut D1 and/or D2 receptors, we first analyzed the influence of DAT hypofunction on the ileal contractile response on basal tone, evoked by the non-cumulative addition of the exogenous monoamine (Figure 1A,B). The addition of dopamine determined a concentration-dependent relaxant response characterized by a reduction of the spontaneous contraction amplitude until complete disappearance, that was significantly higher in isolated ileal segments from DAT^+/−^ mice compared to WT animals (for DAT^+/−^ mice Emax = −288 ± 3 vs. Emax = −187 ± 5 for WT mice, *p* < 0.001; Figure 1A,B). To characterize the involvement of D1 and D2 receptors in the dopamine-induced effects, the response to 30 µM dopamine was evaluated in absence or presence of the D1 receptor antagonist, SCH23390, or the D2 receptor antagonist, sulpiride, in ileal preparations from WT and DAT^+/−^, precontracted with 10 μΜ CCh. SCH23390 significantly reduced dopamine-mediated relaxation of ileal segments from both WT and DAT^+/−^ mice (−32% *p* < 0.01 and −55% *p* < 0.001, respectively), whereas sulpiride, a D2 receptor antagonist, did not affect the dopamine response in both groups (Figure 1C). D1 receptor immunoreactivity was then evaluated in the LMMPs from WT and DAT^+/−^ mice, resulting comparable in both genotypes (Figure 1D,E).

### 3.2. DAT Heterozygosis Affects EFS-Evoked Neuromuscular Response of Small Intestine

**Figure 2:**
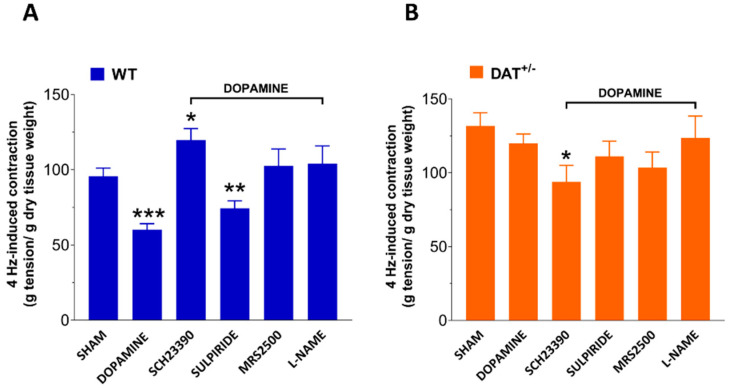
DAT hypofunction affects dopaminergic neuromuscular response. Neuromuscular excitatory response induced by 4 Hz-EFS in absence or presence of 10 µM dopamine after pretreatment with SCH23390, sulpiride, MRS2500 or L-NAME in isolated ileal preparations of WT (**A**) and DAT^+/−^ (**B**) mice. Data are reported as mean ± SEM. * *p* < 0.05, ** *p* < 0.01, *** *p* < 0.001 vs. respective control mice. *n* = 5 mice/group.

To evaluate the effect of dopamine on neurally evoked cholinergic contractions, the response to 4-Hz-EFS was assessed with or without a 2-min-preincubation with 10 μM dopamine. In DAT^+/−^ mice, 4-Hz-EFS determined a 1.3-fold increase of contraction compared to WT mice. Pretreatment with 10 µM dopamine reduced the neuromuscular response only in ileal segments from WT mice (−36%, *p* < 0.001; Figure 2). To further investigate which dopamine receptor subtype is responsible for the higher neuromuscular response in ileal segments from DAT^+/−^ mice, the effect of SCH23390 or sulpiride on 4-Hz-EFS-mediated cholinergic contraction was evaluated. As shown in Figure 2, preincubation with SCH-23390 in presence of 10 µM dopamine caused a significant increase of the 4-Hz-induced contraction (*p* < 0.05) in ileal preparations from WT mice whereas in DAT^+/−^ mice produced a significant reduction (*p* < 0.05). Following preincubation with sulpiride, dopamine caused a significant reduction of the neuromuscular response in ileal preparations from WT mice (*p* < 0.01) but not in those from DAT^+/−^ mice (Figure 2). Since DAT^+/−^ showed a higher dopamine-induced relaxation mainly mediated by D1 receptor activity, we tested whether other neurotransmitter pathways, such as purinergic and nitrergic pathways, were involved in the altered EFS-evoked neuromuscular response of ileal preparation from DAT^+/−^ mice. Intriguingly, the preincubation with MRS2500 or L-NAME in presence of dopamine did not affect the neuromuscular response in both the genotypes (Figure 2). 

### 3.3. DAT Hypofunction Does Not Affect Nitrergic Neurotransmission

**Figure 3:**
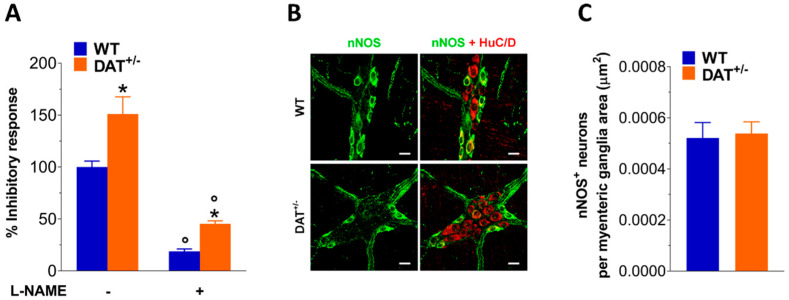
DAT influences NO-mediated relaxation. (**A**) 10 Hz EFS-evoked relaxation in NANC conditions with or without or 100 μM L-NAME (pan-NOS inhibitor) in ileal segments of WT and DAT^+/−^ mice. (**B**) Representative confocal microphotographs showing the distribution of HuC/D (red) and nNOS (green) and (**C**) analysis of nNOS^+^ neurons in ileal LMMPs of WT and DAT^+/−^ mice (bars = 22 μm). Data are reported as mean ± SEM. * *p* < 0.05 vs. WT; ° *p* < 0.05 vs. respective control in NANC condition. *n* = 5 mice/group.

To assess the contribution of nitrergic neurotransmission on DAT^+/−^ enteric dysmotility, we tested NO-mediated relaxation in NANC conditions. Indeed, as shown in Figure 3A, EFS NANC-relaxations at 10 Hz were significantly increased in ileal preparation from DAT^+/−^ mice (+ 50%, *p* < 0.05) compared to WT mice. Pretreatment with the pan-NOS inhibitor L-NAME almost completely blocked EFS-evoked NANC relaxation in WT mice. Conversely, in DAT^+/−^ mice, the response was only partially abolished by L-NAME, underlining the presence of a higher dopaminergic tone, with no changes in the number of nNOS^+^ neurons in both genotypes (Figure 3).

### 3.4. DAT Hypofunction Alters Neuromuscular Excitatory Neurotransmission

To further investigate changes in the excitatory neuromuscular response due to DAT genetic reduction, cumulative concentration–response curves to the non-selective cholinergic agonist, carbachol, were performed. Ileal segments from WT and DAT^+/−^ mice displayed comparable responses to higher concentration of carbachol (Supplementary Figure S1A).

**Figure 4:**
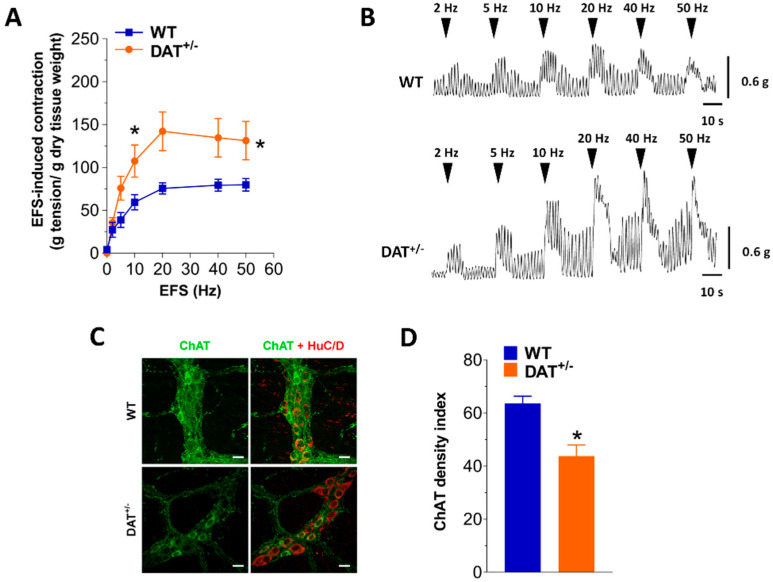
DAT hypofunction influences neuromuscular response. (**A**) Neuromuscular excitatory response induced by EFS (0–50 Hz) in isolated ileal preparations of WT and DAT^+/−^ mice. (**B**) Representative tracings of responses induced by EFS in WT and DAT^+/−^ preparations. (**C**) Representative confocal microphotographs showing the distribution of ChAT (green, marker for cholinergic neurons) and HuC/D (red, pan-neuronal marker) and (**D**) ChAT density index in LMMP preparations of WT and DAT^+/−^ mice. Scale bars = 22 μm. Data are reported as mean ± SEM. * *p* < 0.05 vs. WT mice. *n* = 5 mice/group.

Small intestinal smooth muscle response to the depolarizing agent KCl (60 mM) was not affected by DAT hypofunction (Supplementary Figure S1B). To confirm that ileal contraction changes in DAT^+/−^ mice were caused by alterations in neuromuscular function, we assessed the effect of EFS at increasing frequencies on ileal preparations. In DAT^+/−^ mice, EFS-elicited contractions triggered a significantly increase of the response by about +78% at 10 Hz and +65% at 50 Hz compared to WT mice (Figure 4A,B), suggesting the presence of an altered excitatory neurotransmission. We have previously shown that in mouse ileum, EFS-mediated contractions to frequencies up to 10 Hz are of neuronal cholinergic origin, being sensitive to both tetrodotoxin and atropine [20,25]. Intriguingly, ChAT immunoreactivity was found significantly reduced in ileal whole mount preparations of DAT^+/−^ mice compared to WT mice (−31%, *p* < 0.05; Figure 4C,D). The higher EFS-mediated contractions together with no changes in CCh- or KCl-mediated contractions and reduced ChAT immunoreactivity in DAT^+/−^ mice might advocate for a contributing role of other excitatory pathways such as the tachykininergic or glutamatergic neurotransmission.

### 3.5. DAT Genetic Reduction Influences Tachykininergic and Glutamatergic Neurotransmission

**Figure 5:**
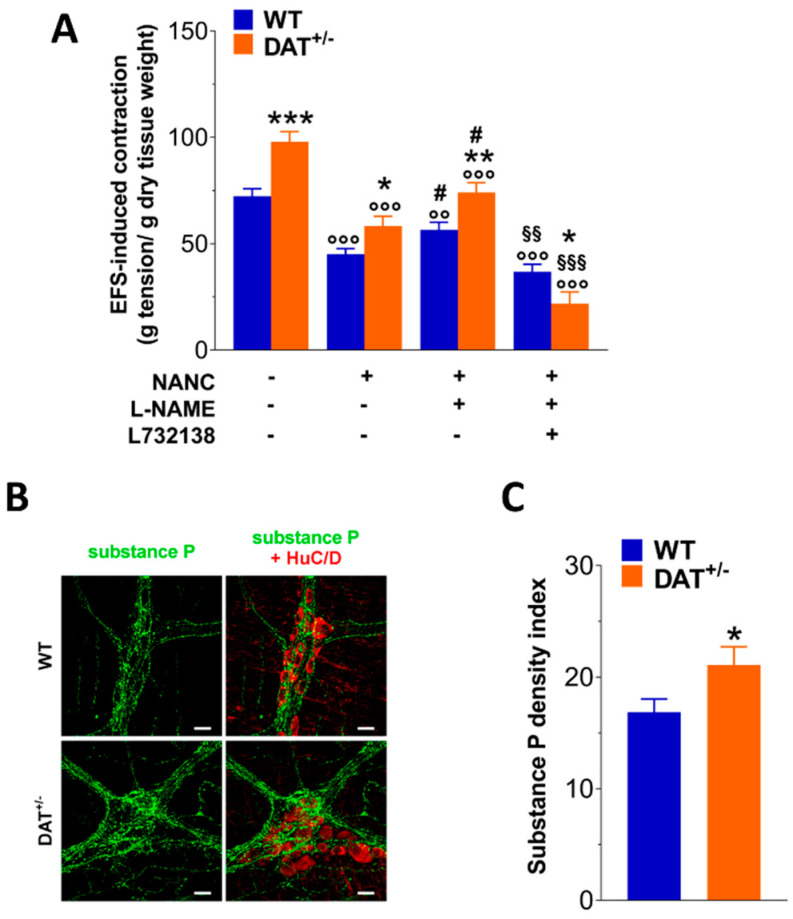
DAT hypofunction influences tachykininergic neurotransmission. (**A**) Tachykininergic nerve-evoked contractions induced by 10 Hz-EFS, in NANC condition with or without L-NAME or L732138 in isolated ileal preparations of WT and DAT^+/−^ mice. (**B**) Representative confocal microphotographs showing the distribution of substance P (green) and HuC/D (red, pan-neuronal marker) and (**C**) substance P density index in LMMP preparations of WT and DAT^+/−^ mice. Scale bars = 22 μm. Data are reported as mean ± SEM. * *p* < 0.05, ** *p* < 0.01, *** *p* < 0.001 vs. WT mice; °° *p* < 0.01, °°° *p* < 0.001 vs. respective control in absence of antagonists; #*p* < 0.05 vs. respective control in NANC condition; §§ *p* < 0.01, §§§ *p* < 0.001 vs. respective control with L-NAME. *n* = 5 mice/group.

Considering the higher EFS-induced contraction together with the higher inhibitory response mediated by dopamine in DAT^+/−^ mice, we decided to evaluate the influence of DAT heterozygosis on tachykininergic and glutamatergic neurotransmissions. 10-Hz-EFS stimulation caused a marked increase of the contraction (+ 35%; *p* < 0.001; Figure 5A) in ileal preparations of DAT^+/−^ mice compared to WT mice. Furthermore, the excitatory response in NANC condition was still higher in DAT^+/−^ mice compared to WT mice (*p* < 0.05; Figure 5A) and the pre-treatment with L-NAME caused a significantly increase of the contraction in both genotypes that resulted higher in DAT^+/−^mice. Intriguingly, the incubation with 10 µM L732128, a neurokinin 1 receptor antagonist, almost completely blocked NANC-mediated neuromuscular contraction in DAT^+/−^ mice (Figure 5A).

**Figure 6:**
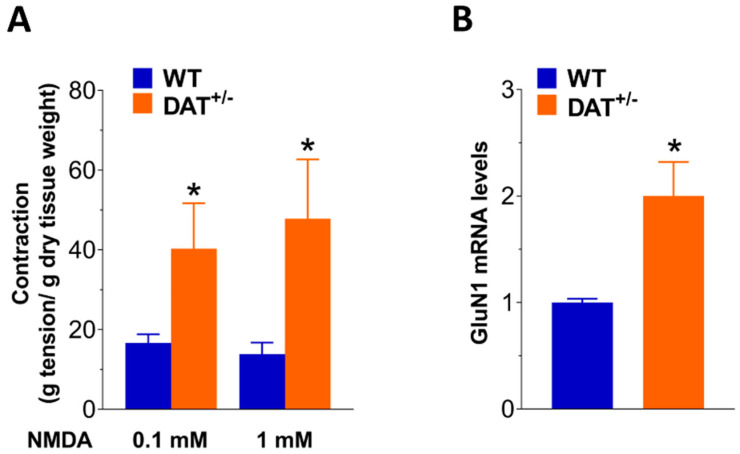
DAT hypofunction influences glutamatergic neurotransmission. (**A**) 0.1 mM and 1 mM NMDA-induced contractions in isolated ileal preparations of WT and DAT^+/−^ mice. (**B**) GluN1 mRNA levels in LMMP preparations of WT and DAT^+/−^ mice. Data are reported as mean ± SEM. * *p* < 0.05 vs. WT mice. *n* = 5 mice/group.

In parallel, immunofluorescence for substance P, a member of the tachykinin family of neuropeptides, with higher affinity for NK1 than for NK2 or NK3 receptors [26], increased about 1.2-fold in the LMMP preparations of DAT^+/−^ mice compared to those obtained from WT animals, corroborating an abnormal tachykininergic neurotransmission in DAT^+/−^ mice (Figure 5B,C). Since NMDA receptors are expressed in the gut and involved in altered bowel motor function and visceral pain sensation in functional gastrointestinal disorders, such as IBS [27,28], we examined the influence of DAT genetic reduction on NMDA neurotransmission, by evaluating the effect of 100 µM and 1 mM NMDA exposure in isolated ileal segments in Mg^++^-free conditions. A marked increase in NMDA-mediated response was observed in ileal preparations from DAT^+/−^ mice, exposed to both concentrations, compared to those from WT mice (*p* < 0.05; Figure 6A). Furthermore, mRNA levels of the ubiquitous and functional subunit of NMDA, GluN1, which plays an important role in inflammation-induced dysmotility and hyperalgesia [29], were significantly higher in LMMP preparations from DAT^+/−^ mice (*p* < 0.05; Figure 6B).

### 3.6. DAT Hypofunction Induces Morphological Abnormalities in the Myenteric Neuroglial Network

**Figure 7:**
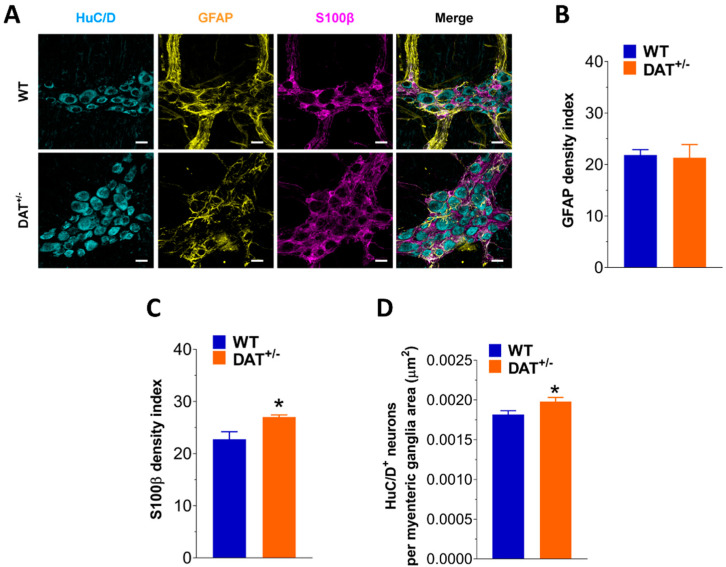
DAT hypofunction affects neuroglial phenotype. (**A**) Representative confocal microphotographs showing the distribution of HuC/D^+^ neurons (cyan), GFAP^+^ (yellow) and S100β^+^ (magenta) glial cells in WT and DAT^+/−^ LMMPs preparations (bars = 22 µm). (**B,C**) Changes in GFAP (**B**) and S100β (**C**) density index in WT and DAT^+/−^ LMMPs preparations. (**D**) Analysis of HuC/D^+^ neurons in ileal LMMPs of WT and DAT^+/−^ mice. Data are reported as mean ± SEM. * *p* < 0.05 vs. WT. *n* = 5 mice/group.

Changes in neurotransmission are known to influence ENS morphology [17,18]. In the myenteric plexus of DAT^+/−^ mice, beside a higher number of HuC/D^+^ neurons, a marked increase of S100β immunofluorescence was found in ileal preparation from DAT^+/−^ mice, by about 18% compared to WT mice, with no changes in GFAP immunoreactivity (Figure 7).

## 4. Discussion

In the gut, dopamine can be produced both by enteric neurons and by non-nervous cells, including the gastrointestinal epithelium [30,31], immune cells [32], and bacteria [33]. Several drugs acting on the dopaminergic system have been used for treating enteric peripheral diseases. Specifically, antidopaminergic gastrointestinal prokinetics (e.g., domperidone, metoclopramide, levosulpiride) are commercially available to alleviate foregut motor disorders, including functional dyspepsia, gastroesophageal reflux disease and gastroparesis [34]. Moreover, changes in DAT availability have been associated to gastrointestinal dysfunctions in Parkinson’s patients [35] as well as in psychiatric disorders [13].

We, here, show, for the first time, that DAT genetic reduction has the following consequences on ENS:

(i) an enhanced motor response primarily mediated by D1 receptors;
(ii) an increased excitatory response mediated mostly by tachykininergic and glutamatergic neurotransmission via NK1 and GluN1 receptors, respectively;
(iii) altered morphology of the myenteric neuroglial network associated to reactive gliosis as well as to changes in cholinergic and tachykininergic neurochemical coding.

Previous studies have shown reduced dopamine content in central neurons as well as in the colon of DAT homozygous mice [3,36,37]. These findings evidence, albeit indirectly, the prominent role of DAT-mediated processes in these tissues and advocate for a not efficient clearance of dopamine from the synaptic cleft in DAT^−/−^ mice, resulting in an altered dopaminergic phenotype [38]. However, even if DAT deficiency can be considered an ideal model for investigating early Parkinson’s disease [39], DAT^−/−^ mice exhibit phenotypic characteristics, such as dwarfism and growth deficit that make them unsuitable for the study of molecular mechanisms underlying gastrointestinal disorders, such as those associated to psychiatric pathologies (e.g., attention-deficit/hyperactivity disorder, schizophrenia or bipolar disorders). Furthermore, these mice have to be nourished with an enriched diet to prevent premature death [40] which might affect their metabolic activity, gut microbial composition and gastrointestinal function [41,42,43]. These critical issues are not encountered with the use of DAT^+/−^ mice, which do not require specific dietary intervention.

To better define the role of enteric dopaminergic neurotransmission on enteric motility [44,45], studies in mice deficient for neuronal dopamine, D2 or D2 + D3 receptors, evidenced that the inhibitory effect of dopamine on the intestinal neuromuscular function likely involves D2 receptors located within the ganglia, where they are implicated in modulating cholinergic neurotransmission and peristalsis [7]. However, in wild type mouse small intestine, more recent findings demonstrated that dopamine causes relaxation and reduction of the spontaneous contractions via activation of D1-like receptors [4,44]. 

In our study, the non-cumulative addition of exogenous dopamine determined a significant reduction of ileal spontaneous amplitude, with no influence on 4-Hz-EFS-induced in DAT^+/−^ animals. In order to evaluate which dopaminergic receptor was involved, we blocked D1 receptors, with SCH-23390 and D2 receptors, with sulpiride, which have shown higher selectivity for dopamine receptors than adrenergic receptors [4]. Indeed, also in the gastrointestinal tract, dopamine can activate adrenergic receptors only at much higher concentrations than those required to activate D1 and D2 receptors. In transgenic animals, dopamine-mediated inhibitory response on 4 Hz-EFS-induced contraction was reduced by pretreating ileal preparations with SCH23390, but not with sulpiride, suggesting the involvement of D1 receptors, which is consistent with a peripheral altered dopaminergic phenotype. In wild type ileal preparations, preincubation with dopamine resulted in almost a 50% decrease of 4-Hz-EFS-mediated contraction, that was completely reverted after D1 receptor blockade, suggesting that the mouse ileum neuromuscular function is controlled by a tonic dopaminergic restraint. Indeed, DAT^−/−^ mice exhibited an enhanced dopamine-mediated inhibitory transmission of colonic preparations [3]. In small intestine preparations obtained from DAT^+/−^ mice, 4-Hz-EFS-induced contractions were insensitive to dopamine pretreatment with or without sulpiride. However, in this experimental group, 4-Hz-EFS-induced contractions in the presence of dopamine were reduced in the presence of SCH23390 suggesting that a D1-mediated hyperdopaminergic transmission may be potentially balanced by an increased excitatory transmission.

Considering that the overall gastrointestinal propulsive motility is known to result from a dynamic integration of circular and longitudinal smooth muscle contractions under strict regulation of both excitatory and inhibitory neurotransmitter pathways [46], the enhanced contraction observed in the presence of dopamine and SCH23390 on 4-Hz-induced contraction could depend upon an altered contribution of other inhibitory receptors, such as the nitrergic and purinergic receptors [20,47,48]. However, this hypothesis can be discarded since incubation with the NO synthase inhibitor, L-NAME or P2Y1 antagonist did not induce any modification of the neurally-evoked cholinergic contraction induced by 4-Hz-EFS in presence or absence of dopamine in ileal segments from DAT^+/−^ mice [4,17,19]. The increased excitatory transmission could be ascribed to a higher cholinergic tone. This hypothesis can be also abandoned since the concentration-response curve to carbachol and the muscular response to 60 mM KCl resulted comparable between genotypes, to highlight that the cholinergic receptors expressed on smooth muscle cells and muscular tissue [20,49] are physiologically intact and are not influenced by DAT expression deficiency. In addition, enhancement of EFS-mediated contractions at frequencies >10 Hz and reduction of ChAT immunoreactivity in DAT^+/−^ mice support the possible involvement of other excitatory pathways such as the tachykininergic and glutamatergic pathways. In DAT^+/−^ mice small intestine, the increase of electrically-evoked tachykininergic contractions in NANC conditions was further confirmed by blockade of NK1 receptor activity. To better interpret these results, we examined the distribution of substance P in ileal specimens by immunohistochemistry, observing a higher immunoreactivity in the myenteric plexus of DAT^+/−^ mice. These data provide the first demonstration of adaptive changes involving the small intestine tachykininergic neurotransmission in response to a genetically driven hyperdopaminergic tone. Accordingly, increased expression of substance P was observed in rat brain and colon after 6-OHDA-induced dopamine depletion [50,51]. The enteric tachykininergic system has a fundamental role in the maintenance of the intestinal neuromuscular function, and alterations in substance P-mediated neurotransmission, i.e., higher content and release from enteric neurons as well as immune cells of intestinal lamina propria, may participate to the pathogenesis of some gastrointestinal disorders of high clinical impact, such as chronic inflammatory diseases [52,53,54]. Several groups have shown in the central nervous system the functional interaction between D1 receptor and NMDA receptors and have suggested that NMDA receptors may modulate D1 receptor-mediated functions, since blockade of NMDA receptor activity reduced the ability of D1 to regulate neuronal activity [55,56,57]. In the myenteric plexus, the levels of GluN1 were upregulated in DAT^+/−^ mice. In the last few years numerous studies have demonstrated that blockade of NMDA receptors may exert gastrointestinal neuroprotective effects against inflammation and glutamate-mediated ischemia/reperfusion injury, advocating for a possible role of endogenous glutamate in both acute and chronic inflammatory conditions [29]. Furthermore, GluN1 subunits, localized to cell bodies in dorsal root ganglion and in peripheral terminals of primary afferents innervating the rat gastrointestinal tract, mediate the local release of neuropeptides, such as CGRP and SP, which play an important role in neurogenic inflammation and hyperalgesia [29]. Interestingly, both glutamate and substance P are described as enteric neurotransmitters involved in the modulation of the enteric motor and sensory functions [28,29,58]. Overall, based on the present findings, it is plausible to hypothesize that upregulation of tachykininergic and glutamatergic enteric neurotransmission, could contribute to the small intestinal motor abnormalities occurring in DAT^+/−^ mice. In view of the pathophysiological relevance of both neurotransmitter pathways in gut neurogenic inflammatory processes, we further focused our attention on the mouse small intestine myenteric neuroglial network by whole-mount immunohistochemistry, which has a crucial role in the development of microinflammatory processes predisposing to neuroplastic adaptive changes. A higher number of HuC/D^+^ neurons together with an increased staining of the glial protein S100β, was observed in DAT^+/−^ mice. Enhanced S100β levels, suggestive of reactive gliosis, have been shown during absence of TLR4 or TLR2 signaling [17,18,19,59], enteric dysbiosis [20], diet-induced obesity [53,54,60,61], impaired mitochondrial respiration, mechanical nerve injury [62]. 

## 5. Conclusions

Mice with unbalanced dopamine transmission, due to genetic DAT dysfunction, have been instrumental for understanding dopamine-related brain disorders. Our findings uncover DAT heterozygosis as a highly valuable model for the comprehension of dopamine-mediated effects in the gastrointestinal tract in both physiological and pathological conditions. These observations may also extend to higher center via the so-called gut–brain axis and help to decipher novel therapeutic strategies in an area of active investigation, represented by central nervous system disorders associated to gastrointestinal dysfunction or vice versa [43,51,63].

## Figures and Tables

**Table 1:** Characteristics of primary and secondary antibodies and their respective dilutions used for immunohistochemistry on ileal whole-mount preparations.

Antibody	Host Species	Dilution	Catalog Number	Source
**Primary Antisera (Clone)**				
HuC/D (16A11)	Mouse biotin-conjugated	1:100	A-21272	Thermo Fisher Scientific (Monza, Italy)
nNOS (polyclonal)	Rabbit	1:100	61–700	Thermo Fisher Scientific
GFAP (polyclonal)	Chicken	1:100	ab4674	Abcam (Cambridge, UK)
S100β (EP1576Y)	Rabbit	1:50	ab52642	Abcam
ChAT (polyclonal)	Goat	1:100	AB144P	Sigma-Aldrich (Milan, Italy)
D1 receptor (polyclonal)	Rabbit	1:50	ab20066	Abcam
Substance P (polyclonal)	Guinea pig	1:50	ab10353	Abcam
Secondary Antisera				
Goat anti-rabbit IgG Alexa 488-conjugated	NA	1:1000	A-11008	Thermo Fisher Scientific
Donkey anti-goat IgY Alexa 555-conjiugated	NA	1:1000	A-21432	Thermo Fisher Scientific
Goat anti-chicken IgY Alexa 555-conjugated	NA	1:1000	A-11039	Thermo Fisher Scientific
Goat anti-guinea pig IgG Alexa Fluor 488-conjugated	NA	1:1000	AB_2534117	Thermo Fisher Scientific
Streptavidin Alexa 555-conjugated	NA-	1:1000	S21381	Thermo Fisher Scientific

## Data Availability

The data presented in this study are available on request from the corresponding author.

## Supplementary Materials

The following are available online at https://www.mdpi.com/article/10.3390/biomedicines9050465/s1, Figure S1: DAT hypofunction does not influence ileal muscular response.
