# Supplementary material for: Dopamine Transporter Genetic Reduction Induces Morpho-Functional Changes in the Enteric Nervous System
Source: Biomedicines. 2021 Apr 24;9(5):465. doi: 10.3390/biomedicines9050465 (PMC8146213; doi:10.3390/biomedicines9050465)
Supplement: Supplementary file 1 [file biomedicines-09-00465-s001.zip › biomedicines-1179454-supplementary.pdf]

SUPPLEMENTARY FIGURE

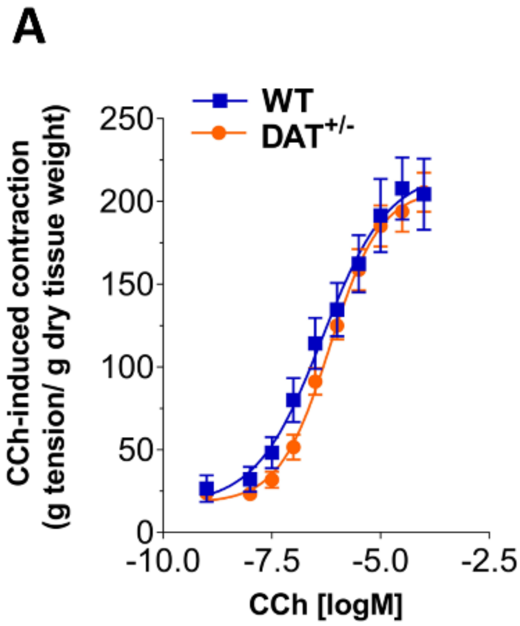

Supplementary Figure S1. DAT hypofunction does not influence ileal muscular response. (**A**) Concentration–response curves to carbachol (CCh), (**B**) KCl-mediated excitatory response in isolated ileal preparations of WT and $DAT^{+/-}$ mice. Data are reported as mean ± SEM.
